# Long-Distance Dispersal by Sea-Drifted Seeds Has Maintained the Global Distribution of *Ipomoea pes-caprae* subsp. *brasiliensis* (Convolvulaceae)

**DOI:** 10.1371/journal.pone.0091836

**Published:** 2014-04-22

**Authors:** Matin Miryeganeh, Koji Takayama, Yoichi Tateishi, Tadashi Kajita

**Affiliations:** 1 Department of Biology, Graduate School of Science, Chiba University, 1-33 Yayoi, Inage, Chiba, Japan; 2 The University Museum, The University of Tokyo, 7-3-1 Hongo, Bunkyo-ku, Tokyo, Japan; 3 Faculty of Education, University of the Ryukyus, 1 Senbaru, Nakagami-gun Okinawa, Japan; Wuhan Botanical Garden, Chinese Academy of Sciences, Wuhan, China, China

## Abstract

*Ipomoea pes-caprae* (Convolvulaceae), a pantropical plant with sea-drifted seeds, is found globally in the littoral areas of tropical and subtropical regions. Unusual long-distance seed dispersal has been believed to be responsible for its extraordinarily wide distribution; however, the actual level of inter-population migration has never been studied. To clarify the level of migration among populations of *I. pes-caprae* across its range, we investigated nucleotide sequence variations by using seven low-copy nuclear markers and 272 samples collected from 34 populations that cover the range of the species. We applied coalescent-based approaches using Bayesian and maximum likelihood methods to assess migration rates, direction of migration, and genetic diversity among five regional populations. Our results showed a high number of migrants among the regional populations of *I. pes-caprae* subsp. *brasiliensis*, which suggests that migration among distant populations was maintained by long-distance seed dispersal across its global range. These results also provide strong evidence for recent trans-oceanic seed dispersal by ocean currents in all three oceanic regions. We also found migration crossing the American continents. Although this is an apparent land barrier for sea-dispersal, migration between populations of the East Pacific and West Atlantic regions was high, perhaps because of trans-isthmus migration via pollen dispersal. Therefore, the migration and gene flow among populations across the vast range of *I. pes-caprae* is maintained not only by seed dispersal by sea-drifted seeds, but also by pollen flow over the American continents. On the other hand, populations of subsp. *pes-caprae* that are restricted to only the northern part of the Indian Ocean region were highly differentiated from subsp. *brasiliensis*. Cryptic barriers that prevented migration by sea dispersal between the ranges of the two subspecies and/or historical differentiation that caused local adaptation to different environmental factors in each region could explain the genetic differentiation between the subspecies.

## Introduction

Seed dispersal is the only way for most land plants to colonize new sites and expand their distribution ranges, because populations of plants are mostly spatially isolated from each other by hundreds of meters or more. For most land plants that have sessile life forms, seed dispersal represents the only way by which populations can exchange individuals and maintain migration and gene flow between distant populations over which pollen cannot move [1], [2]. Thus, “long-distance” seed dispersal (defined by Cain *et al.* [2] as dispersal >100 m) plays an essential role in maintaining connectivity among plant populations and in the cohesiveness of most widespread species.

One of the most effective modes of long-distance dispersal of seeds is sea dispersal, which can disperse seeds >100 km [3]. Because of its effectiveness across long distances, species that exhibit sea dispersal have very wide ranges, such as those usually seen in the species of mangrove trees [4]. Most mangroves have seeds or propagules that can drift on seawater and be transported by ocean currents. Although they have wide distribution ranges, they are still limited at the broadest scales. Various mangrove species from different families distribute only either in the Indo-West Pacific (IWP) or Atlantic East-Pacific (AEP) regions [4]. These two biogeographic regions are common for many mangrove species and other sea-dispersed plants, indicating that the East Pacific Ocean and African continent have worked as important barriers for seed dispersal for most species of widespread sea-dispersed plants [4], [5]. However, there are a few groups of sea-dispersed plants that have overcome those barriers and have a global distribution range.

Pantropical plants with sea-drifted seeds [6], [7], [8] have an extraordinarily wide range of distribution in littoral areas of the tropics and subtropics worldwide. Members of this plant group are littoral plants and can disperse their seeds over very long distances using ocean currents as a vector. A few species from divergent families are known in this plant group, including *Ipomoea pes-caprae* (L.) R. Br. (Convolvulaceae), *Canavalia rosea* Sweet. (Fabaceae), *Vigna marina* (Burm.) Merr. - *Vigna luteola* (Jacq.) Benth. (Fabaceae), and *Hibiscus tiliaceus* L. - *Hibiscus pernambucensis* Arruda (Malvaceae). All species in this group are believed to have developed mechanisms for long-distance seed dispersal by ocean currents. *Ipomoea pes-caprae* has among the widest ranges of these species.

*Ipomoea pes-caprae* (L.) R. Br., commonly known as beach morning glory, is a creeping vine found on beaches throughout the tropical and subtropical regions of the world [9], [10]. The species has two subspecies, *Ipomoea pes-caprae* subsp. *pes-caprae* and *Ipomoea pes-caprae* subsp. *brasiliensis* (L.) van Ooststr, which are morphologically different in the shape of the leaves and the dimensions of the calyx and corolla [9], [10]. The distribution ranges of the two subspecies are well separated: subsp. *pes-caprae* is only distributed in the northern part of the Indian Ocean (from the Arabian Peninsula to Indonesia) and subsp. *brasiliensis* is found in tropical and sub-tropical areas worldwide, except for the northern Indian Ocean [9]. This is one of the largest ranges of a single species of land plants.

How *I. pes-caprae* has achieved and maintained this extraordinarily wide range around the globe was the first question for this study. The circumglobal range of the species distribution includes several possible land and ocean barriers [5]. Although *Ipomoea pes-caprae* subsp. *brasiliensis* uses a wide range of insect pollinators, gene flow by pollen is not likely between two neighboring but distant populations when they are separated by hundreds of kilometers of ocean (for example, between main islands and an oceanic island). Sea dispersal by drifted seeds would be the only possible way to keep migration between the two populations, and, indeed, the species has seeds that can float on seawater for more than 90 days [11]. However, there are no empirical data to explain how a single species can migrate over such an extremely wide range. Genetic differentiation between *I. pes-caprae* subsp. *pes-caprae* and subsp. *brasiliensis* was the second question. Taxonomists have considered that the morphological differences between the two are at the subspecies level [9], [10], but the distribution boundary between the two subspecies is in a location where no apparent land or oceanic barriers exist. Our recent phylogeographic study using two nucleotide genes [12], Waxy and HSP-90, suggested the presence of a genetic structure that might correspond to IWP and AEP, but the structure was only discussed with regard to the superficial distribution of haplotypes of only two genes. Levels of migration and genetic differentiation between the populations have not been studied yet.

To answer these questions, we tested whether 1. Long distance trans-oceanic dispersal of seeds results in low genetic divergence among continents, 2. Known biogeographic barriers such as continents and boundaries in ocean currents correspond to patterns of genetic differentiation, and 3. Genetic differentiation within subspecies is lower than genetic differentiation between subspecies. We used nucleotide sequences of seven low-copy nuclear markers and performed a coalescent-based approach using Bayesian and maximum likelihood inference. This is a powerful method to demonstrate the amount and direction of migration among populations and is useful to determine the geographic structure of the species over its global range. We studied 272 samples of *I. pes-caprae*, including both subspecies, collected from 34 populations that cover much of the global range of the species.

## Materials and Methods

### Population sampling and DNA extraction

**Figure 1:**
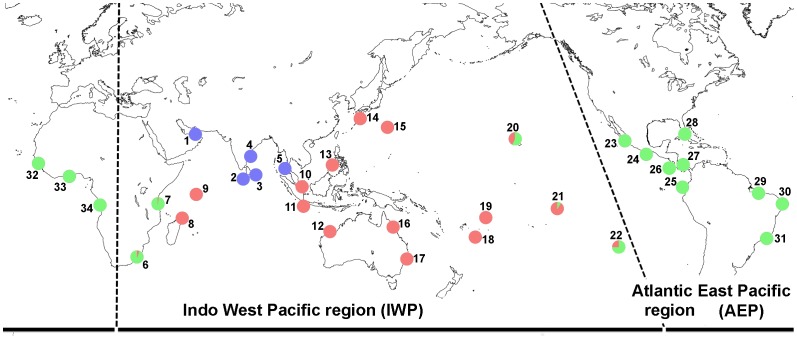
Geographical distributions of the 34 sampled populations used in this study. Detailed information on the populations is given in Table S1. Pie charts suggest the STRUCTURE-derived ancestry of each population based on the coloring used in Figure 3.

Leaf samples of 272 individuals from 34 populations of *I. pes-caprae* were collected across the range of this species (Table S1). Five of these populations (40 individuals; Populations 1–5) were of subsp. *pes-caprae* from the northern part of the Indian Oceanic region and the rest were of subsp. *brasiliensis*. We selected populations that provided good coverage of the global distribution range of the species. The locations of the 34 sampled populations are shown in Figure 1. Field work to collect samples was performed between 1999 and 2008. No specific permissions were required to access the sampling sites that were neither privately owned nor protected in any way. The species studied is not endangered, but is rather a weed, and was not protected at the time of collections. Since *I. pes-caprae* is a vine and individuals can spread for over 20 meters on beaches, we carefully kept a large distance between selected individuals to avoid multiple samples from the same individual. Leaf samples were kept and dried in silica-gel. Voucher specimens of these samples were deposited in the Herbarium of the University of Ryukyus (URO).

Total DNA was isolated from silica-gel leaf pellets using the cetyltrimethyl ammonium bromide (CTAB) extraction method as was described in a previous study [7], with the addition of purifying the DNA solution with glass powder using GeneCleanIII (Bio 101, Vista, CA, USA).

### Locus selection, polymerase chain reaction (PCR), and sequencing

We searched the target loci from randomly chosen low-copy genes and ESTs in published papers and a DNA database. In the first step to search for candidate markers, we randomly chose 14 single or low-copy genes identified in *Ipomoea* and other species from the published literature as candidate loci for PCR amplification: Beta-amylase (Primers 3F and 4R) [13], Chs (Primers 52F and 1198R) [14], Adh, Cam, G3pdh, Tpi, Aat, Chi, and Pgi [15], Waxy [16], SAMT (Primers F3 and R2) [17], HSP-90 [18], ALS and ANS [19]; as well as 6 loci from *Ipomoea batata* EST data in GenBank. We then obtained PCR primers from the literature, or designed primers from the EST sequence data in GenBank. We performed PCR amplification for the 20 loci using a sample of *I. pes-caprae* subsp. *brasiliensis* collected from the Philippines and selected seven loci that produced only a single band. All other loci that produced multiple bands or no bands were not considered further in this study. In the second step, we tested whether clear sequence data could be obtained for the seven candidate markers by the direct sequencing method even though we were using samples of subsp. *pes*-*caprae* and subsp. *brasiliensis* from around the world. We used 12 individuals from 6 populations that well covered the range of subsp. *brasiliensis*: West Atlantic (Panama-Colon), East Atlantic (Ghana), West Pacific (Philippine), East Pacific (Panama-Vera Cruz), Indian Ocean (Western Australia) and a population of subsp. *pes-caprae* (UAE). All the seven loci yielded clear sequence data for direct sequencing using an ABI 3500 automated sequencer (Applied Biosystems, Foster City, CA, USA). In the last step, to make sure that there was no variation within primer regions, we designed internal specific primers for all seven loci (Table S2) based on conserved regions in the alignment that included the 12 samples of *I. pes-caprae* as well as all possible relevant sequences from closely related *Ipomoea* species obtained from GenBank. We used all seven loci as markers in this study. They were: *Waxy* gene encoding granule-bound starch synthase I (GBSSI), chalcone synthase gene (*Chs*), triosephosphate isomerase gene (*Tpi*), heat shock protein 90 (*Hsp-90*), anthocyanidin synthase (*ANS*), acetolactate synthase (*ALS*), and 1 gene from the EST database of the NCBI (*EST*). For three of the seven loci (*ALS*, *TPI*, and *EST*), all sequenced regions were in coding regions, while for the other 4 (*ANS*, *Chs*, *HSP-90*, and *Waxy*) sequenced regions included both coding and noncoding regions (Table S2). Direct sequencing analyses for all of the samples for the seven markers produced clear sequence profiles. Although double peaks would be expected for possible heterozygotes in each marker, no overlapping peaks were detected for any markers in this study.

In all of the experiments explained earlier, PCR was performed in reaction volumes of reaction mixtures (30 µL/reaction) containing c. 10–30 ng of template DNA, 0.12 µL (0.6 units) of ExTaq DNA polymerase (TaKaRa Bio INC, Otsu, Shiga, Japan), 3 µL of ExTaq PCR buffer [10 mmol/L Tris-HCL (pH 8.3), 50 mmol/L KCL, 1.5 mmol/L MgCl_2_], 2.4 µL of 0.2 mmol/L dNTP solution, 2.4 µL of 2.0 mM MgCl_2_, and 0.8 µL of 10 pmol/L for each of 2 primers. The PCR conditions were as follows: 3 min for initial denaturation at 95°C, followed by 35 amplification cycles of 1 min denaturation at 95°C, 1.5 min annealing at a fragment specific temperature, 1 min extension at 72°C, and a final 10 min extension at 72°C. We used the annealing temperature of 54°C for all primers. The PCR products were visualized by 0.8% agarose gel electrophoresis with ethidium bromide staining, and amplified DNA was purified using ExoSAP-IT (USB Corp., Cleveland, Ohio, USA), according to the manufacturer's instructions. The cycle sequencing reactions were carried out using the ABI BigDye Terminator version 3.1 Cycle Sequencing Kit (Applied Biosystems), and the sequencing reaction products were purified by ethanol precipitation. The purified PCR products were sequenced directly on an ABI 3500 automated sequencer (Applied Biosystems, Foster City, CA, USA).

We determined nucleotide sequences of population samples for five out of the seven markers, the exceptions being *Waxy* and *HSP-90*. All the obtained sequences were visually edited and aligned by SEQSCAPE V2.5 software (Applied Biosystems). All new sequence data obtained in this study were deposited in GenBank (accession nos. KF296474-KF296517 and KF296525-KF296536). For further population genetic studies, we also used the sequence data for *Waxy* and *HSP-90* (GenBank accession nos. KF296518-24 and KF296558-62) which had been published in our previous study [12].

### Nucleotide diversity and neutrality tests

Sequences were aligned using BioEdit v. 5.0.9.1 [20]. Indels (insertions and deletions) were excluded from all analyses in this study because some of the analyses do not allow the presence of indels, and we found many fewer indels than nucleotide substitutions, so removing them did not have a large effect on the outcome of the analyses. Coding regions and open reading frames were identified in comparison with the *Ipomoea batata* EST data from GenBank. To determine the phylogeographic relationship among populations, a statistical parsimony network was inferred for each locus using TCS1.06 [21]. For each locus and for each pair of populations, the number of segregating sites (S); the extent of nucleotide polymorphism in terms of θw [22]; and the nucleotide diversity in terms of π [23] at total sites (πt), silent sites (πs), and nonsynonymous sites (πa) were estimated using DnaSP v. 5.10.01 [24]. Each locus was tested for departures from neutral expectations using Tajima's D [25] and Fu and Li's D* and F* [26] statistics in DnaSP because the coalescent-based approaches employed in this study basically assume the neutrality of markers.

### Intra-locus recombination

Recombination is believed to be common in nuclear loci and to influence the results of genealogy-based analyses [27]. Therefore, we inferred recombination events with the “four-gamete test” using DnaSP ver. 5.10.01. Although this test only evaluates the minimum number of intra-locus recombination events, it is sensitive to the presence of recombination. When the test suggested an intra-locus recombination for a marker locus, we discarded the minimum block that contained recombination and retained the longest possible sequence without recombination for subsequent analyses (Table S2). We then assumed in the following analyses that all sequence data obtained from each locus shared the same genealogical history.

### Population structure

The genetic structure of *I. pes-caprae* was investigated using a model-based clustering algorithm implemented in STRUCTURE V. 2.3 [28]. This program employs a Bayesian algorithm to infer the true number of clusters (K) in a sample of individuals and to group them into the most likely number of clusters (K) that maximizes the within-cluster Hardy–Weinberg and linkage equilibrium. A total of 20 replicate runs were conducted for every value of K between 1 and 8, with a burn-in of 50,000 iterations and a run length of 500,000 iterations. An admixture model was used without prior population information. STRUCTURE analyses were performed on all 34 populations. The most likely number of clusters (K) was evaluated using ΔK [29], which is an ad hoc statistic based on the rate of change in the log probability of data between successive K values. The K value chosen was the one that gave the highest value of ΔK.

We also calculated population pairwise Fst [30] to show the level of genetic differentiation between populations within regions using arlesumstat in Arlequin ver. 3.5 [31]. The calculation was first done for each locus separately, and then the average values for all seven loci were obtained.

### Migration rates between oceanic regions

To assess migration rates, the direction of migration, and the genetic diversity among populations of *I. pes-caprae*, we applied a coalescent-based approach [32], [33] using the Bayesian and maximum likelihood inference methods implemented in MIGRATE-N version 3.3.1 [34], [35], [36]. The neutrality of sequence data was tested prior to the analyses. All populations were pooled into five regional groups: Indian Ocean (IO), West Pacific (WP), East Pacific (EP), West Atlantic (WA), and East Atlantic (EA), separated by three land barriers (the African continent, Malay Peninsula, and American continents) and two oceanic barriers (East Pacific Ocean and Atlantic Ocean) that are known geographic barriers for widespread sea-dispersal plants [5]. In addition, populations of subspecies *pes-caprae* from the northern part of the Indian Ocean were included as a group into the analyses. According to the linear setting of the five regional groups, we employed the stepping-stone migration model with asymmetric rates [37]. For each of the five regional groups, the genetic diversity in terms of the mutation-scaled effective population size (θ = 4 Neµ, where Ne is the effective population size and µ is the mutation rate per site per generation), migration rate (M = m/µ, where m is the rate of migration for each locus) and number of migrants per generation (Nm = Mθ/4) were estimated. These parameters were also estimated for subsp. *pes-caprae*, but the migration rate involving subsp. *pes-caprae* was estimated between the subspecies and all other samples of subsp. *brasiliensis*. Starting parameters for migrant values and θ were generated from a few trial runs. Bayesian MCMC coalescent modeling was used to provide parameter estimates based on full likelihood estimation and the decreased computation time of the approximation in comparison to maximum likelihood estimates. Bayesian parameters included an update frequency of 0.5, a Metropolis–Hastings sampling algorithm for both θ and M; uniform priors were placed on θ from 0 to 0.002 and M from 0 to 5000. Starting parameters for migrant values and θ were generated from the first few trial runs. An adaptive heating scheme with four chains and a swapping interval of one was applied. We used the Felsenstein 84 model of evolution, and set the transition to transversion ratio to two as default values in the program. Six independent MCMC runs of varying length and burn-in were conducted which produced similar results. Hence, we presented mean results of all runs. Using tracer v1.5 [38] convergence of the likelihood in MCMC chains and effective sample size (ESS) were observed following the burn-in. The analyses were considered as converged upon a stationary distribution if the different runs generated similar posterior density distributions with a minimum ESS of 100 [39], [40]. For maximum likelihood analysis, 20 short chains (length 5.0×10^4^) followed by five long chains (length 5.0×10^5^) with a sample increment of 100 for both runs were conducted, and the first 15,000 generations were discarded as burn-in at the beginning of each chain. An adaptive heating scheme with four chains and a swapping interval of one was applied. Maximum likelihood estimates were verified with three replicate Markov Chain Monte Carlo (MCMC) simulation runs to ensure the convergence of similar values for θ.

## Results

### Nucleotide variation and neutrality tests

A total of seven nuclear loci were sequenced for 272 individuals representing 34 populations of *I. pes-caprae. ANS*, *HSP-90*, *TPI*, and *Waxy* included five, one, three, and one indel sites, respectively, and those indels were excluded from further analyses. The aligned length of the nucleotide sequences ranged from 425 to 785 bp with a total length of 4075 bp after excluding gaps and recombination sites (Table S2). The nucleotide diversity at each locus and over the seven loci for each pair of populations are presented in Table 1, S3, and S4. The levels of polymorphism differed between loci; *Chs* was the most polymorphic (k = 8.721) and *Waxy* the least (k = 1.140) (Table 1). The levels of nucleotide polymorphism (πt) over the seven loci were higher in subsp. *brasiliensis* (πt = 0.0068) than in subsp. *pes-caprae* (πt = 0.0027). The ratio of synonymous to non-synonymous polymorphism was compared within and between populations. We did not find any fixed non-synonymous mutations within a population. The diversity at noncoding and synonymous sites was higher than that at nonsynonymous sites (Table 1). The divergences between the two subspecies were larger (K_t_ = 0.0098) than the other comparisons between groups, which were K_t_ = 0.0054 between the East Atlantic and Indian Oceans, K_t_ = 0.0037 between the Indian and West Pacific, and K_t_ = 0.0021 between the East Pacific and West Atlantic (Table S3).

**Table 1:** Summary of polymorphisms for the studied loci.

					Nucleotide diversity				
locus	length (bp)	K	H	Hd	πtotal	πs	πa	πa/πs	πnc
*ALS*	430	4.198	13	0.8	0.01	0.006	0.002	0.38	-
*ANS*	680	4.452	15	0.9	0.01	0.005	0.001	0.20	0.007
*Chs*	738	8.721	10	0.7	0.01	0.026	0.008	0.31	0.006
*EST*	425	0.927	9	0.7	0	0.004	0.002	0.63	-
*TPI*	443	1.477	12	0.8	0	0.008	0.004	0.46	-
*HSP* *	785	1.264	7	0.8	0	0.008	0.001	0.09	0.007
*Waxy* *	574	1.140	5	0.6	0	0.002	0.001	0.40	0.001

Number of polymorphism (K), Number of haplotypes (H), Haplotype diversity (Hd), Nucleotide diversity of all sites (πtotal), that of noncoding regions (πnc), synonymous (πs) and nonsynonymous sites (πa) calculated by DnaSP.

* Suggest the data was obtained from a previous study (Miryeganeh et al. (in press)).

### Haplotype network

**Figure 2:**
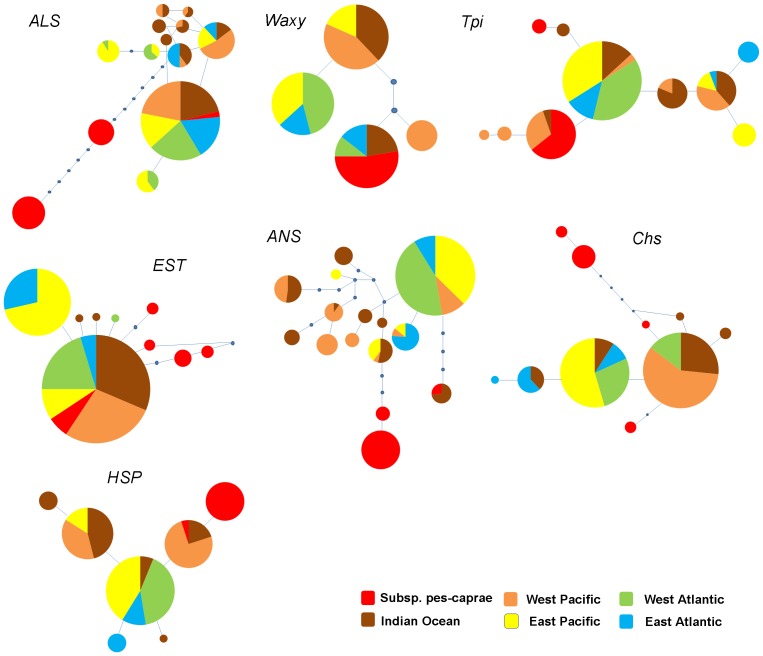
Parsimony networks of haplotypes of *Ipomoea pes-caprae* from 7 nuclear loci. Each circle shows different haplotypes and each color represents haplotypes from different geographical regions. Red represents haplotypes of subsp. *pes-caprae*. Brown, orange, yellow, green, and blue represent subsp. *brasiliensis* from the Indian Ocean, West Pacific, East Pacific, West Atlantic, and East Atlantic, respectively.

The relationships among the haplotypes are shown in parsimony networks (Figure 2). Subspecies *pes-caprae* had at least one unique haplotype separated from subspecies *brasiliensis* with two to 20 steps, but also at least one haplotype was shared between the two subspecies except for *Chs*. For subsp. *brasiliensis*, there was at least one haplotype in all of the loci shared between the populations of the Indian Ocean and West Pacific regions, between the West and East Atlantic regions, and between the Atlantic and East Pacific regions. In three cases (*ALS*, *Tpi*, and *EST*) networks possessed one main haplotype shared among all populations of geographical regions, and in two cases (*ALS* and *EST*) haplotypes were evenly shared between the two subspecies. In the remaining four loci, there were at least two major close haplotypes that were shared in all geographical regions (except for *ANS*, where the haplotype from the Indian Ocean was separated from the others). In order to visualize the degree of haplotype endemicity and haplotype sharing among regional populations, we used pie charts in the haplotype network (Figure 2) and plotted the distribution of haplotypes on maps (Figure S1).

### Genetic differentiation and population structure

**Figure 3:**
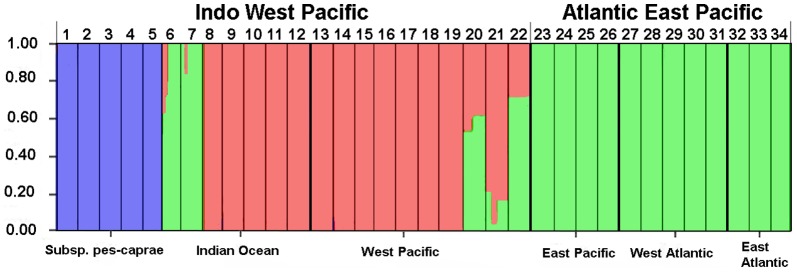
STRUCTURE analysis of *Ipomoea pes-caprae* populations based on 7 nuclear markers. Vertical bars represent the membership coefficients (Q) of individual plants when K = 3. The horizontal axes correspond to the regional grouping of populations. Left to right: subsp. *pes-caprae*, Indian Ocean, West Pacific, East Pacific, West Atlantic and East Atlantic regions. Numbers on the upper side designate the population numbers as shown in Figure 1 and Table S1.

The results of assessment of the population structure based on Bayesian likelihood estimates (using STRUCTURE) are shown in Figure 3. The highest likelihood was found for K = 3 and the likelihood values were consistent among the 10 replicate runs. All individuals of subsp. *pes-caprae* were exclusively grouped in a different cluster from subsp. *brasiliensis*. For subsp. *brasiliensis*, samples of IWP populations and AEP populations were almost grouped in different clusters, but the genetic differentiation between the regions was not completely conserved in our geographical setting (Figure 3). In K>3, unclear geographic structures were detected between populations (Figure S3).

The results of population pairwise Fst also suggested a similar pattern of population differentiation and relatively less differentiation among populations within regions (Table S4).

### Migration rates between regional populations

**Figure 4:**
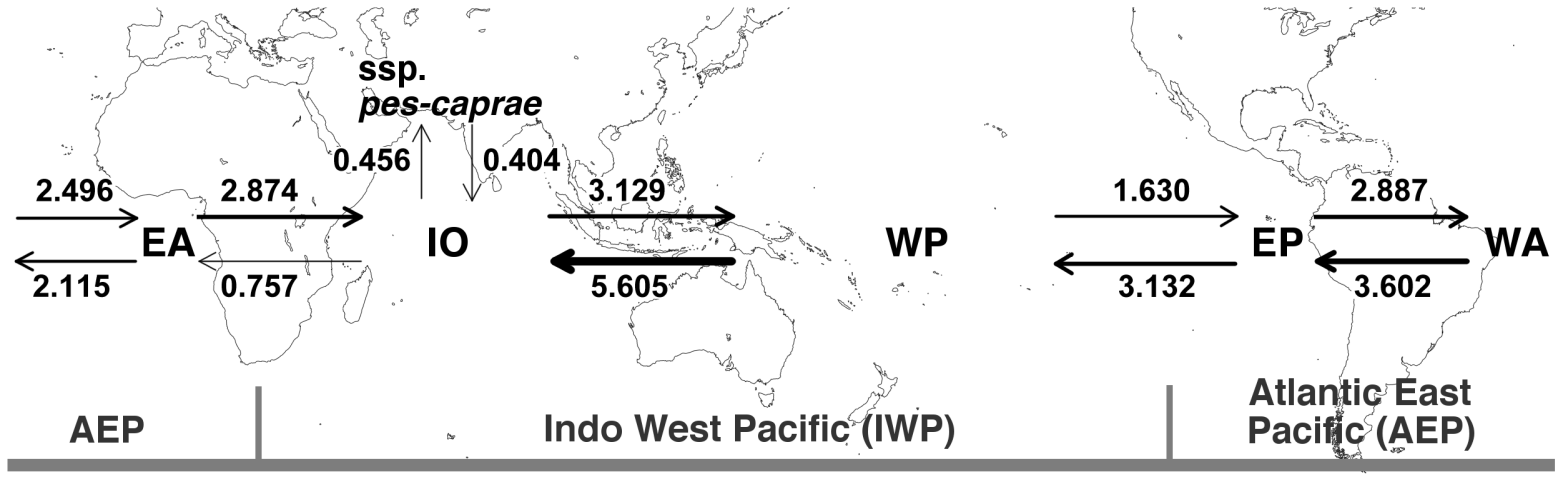
Number of migrants per generation (Nm) between different regional populations. Nm were calculated by a Bayesian method implementation in MIGRATE-N. Regional populations used are East Pacific (EP), West Atlantic (WA), East Atlantic (EA), Indian Ocean (IO), and West Pacific (WP).

Coalescent-based Bayesian estimates by MIGRATE-N indicate asymmetric migration among the *I. pes-caprae* populations (Table 2). The Bayesian estimates showed that genetic diversity was highest in the West Pacific region (θ**_WP_** = 0.00625), and lowest in the East Atlantic region (θ**_EA_** = 0.00300). The most probable estimates of migration rates (M) ranged from 360.0 to 4993.7, with the highest migration observed from the West Pacific to the Indian Oceanic region (M = 4993.7). In contrast, estimates of migration rates between the two subspecies (M = 360.0 and M = 430.0) were lower. The migration between populations was asymmetrical, as is shown by the number of migrants (Nm) in the Table 2 and Figure 4. The maximum-likelihood estimation of migration rates showed almost equal patterns with the Bayesian estimation (Table S5).

**Table 2:** Bayesian estimates of genetic diversity (θ), Migration rates (M) and number of migrants per generation (Nm) obtained by MIGRATE-N.

	Mean (95% CIs)	Nm = Mθ/4	
Θ***_pes-aprae_***	0.00424 (0.00339–0.00493)		
Θ**_IO_**	0.00449 (0.00356–0.00541)		
Θ**_WP_**	0.00625 (0.00528–0.00679)		
Θ**_EP_**	0.00437 (0.00356–0.00507)		
Θ**_WA_**	0.00384 (0.00301–0.00487)		
Θ**_EA_**	0.00300 (0.00190–0.00380)		
**M ** ***_pescaprae_***_**>*brasiliensis***_	360.0 (243.5–410.12)	0.404	
**M ** ***_brasiliensis_***_**>*pescaprae***_	430.0 (290.66–559.3)	0.456	
**M _IO >WP_**	2002.5 (1873.3–3676.7)	3.129	
**M _WP > IO_**	4993.7 (2756.3–5920.0)	5.605	
**M _WP > EP_**	1491.8 (786.7–2123.3)	1.630	
**M _EP > WP_**	2004.2 (1850.0–3676.7)	3.132	
**M _EP > WA_**	3006.8 (2260.0–4826.7)	2.887	
**M _WA > EP_**	3296.9 (2256.7–4620.0)	3.602	
**M _WA > EA_**	3328.8 (2493.3–4183.3)	2.496	
**M _EA > WA_**	2202.7 (1850.0–3676.7)	2.115	
**M _EA >IO_**	2560.5 (1916.7–4113.3)	2.874	
**M _IO > EA_**	1009.9 (680.0–1673.3)	0.757	

WA: West Atlantic, EA: East Atlantic, IO: Indian Ocean, WP: West Pacific, EP: East Pacific. θ = 4 Neµ, where Ne is the effective population size and µ is the mutation rate per site per generation. M = m/µ, where m is the rate of migration for each locus. Nm is number of migrants per generation (Nm = Mθ/4).

## Discussion

### Long distance trans-oceanic dispersal of seeds results in low genetic divergence among continents

Our results using a coalescent-based approach in MIGRATE-N suggest that there is a significant level of migration among populations of *I. pes-caprae* subsp. *brasiliensis* throughout its global range (Figure 4 and Table 2). Although the number of migrant per generation (Nm) between adjacent regional populations is asymmetric, a total Nm of at least 3.6 is maintained between all studied pairs of regional populations. Under the stepping stone model, populations will be strongly differentiated if Nm<1 and will be panmictic if Nm>4 [41]. The values of Nm obtained in this study suggest that subsp. *brasiliensis* has maintained substantial migration throughout its range. Migration among distant regional populations is also suggested by the haplotype networks (Figure 2), in which at least one major haplotype in each gene tree showed a distribution of the haplotype in more than three geographic regions. Because the regional populations studied here, except for those separated by the American continents, are separated by barriers more than 1,000 km wide (Africa and the East Pacific Ocean) that have shaped the genetic structure of many sea-dispersal species [5], long-distance dispersal by sea-drifted seeds is the only way to maintain migration among very distant populations of the subsp. *brasiliensis* connected by the ocean.

Another important finding from this study is the unexpectedly high migration over the American continents (Figure 4 and Table 2), which also helped to maintain the global migration of subsp. *brasiliensis*. Landmasses such as the American continents are apparent barriers for sea-dispersal, because they physically prevent the drifting of seeds. Their strong effects as a barrier to sea dispersal have been documented in some widespread plants in AEP by cpDNA markers, for example, *Hibiscus pernambucensis* [6], [7], *Rhizophora mangle* and *R. racemosa* [42], and *Avicennia germinans* [43]. All of these studies showed a distinctive genetic structure of cpDNA markers over the American continents, and it was suggested that the genetic structure has been maintained at least since the formation of the Isthmus of Panama about 3.1–3.5 million years ago. However, our study suggested a high level of migration (total Nm = 6.489 in Figure 4) over the American continents, even higher than the rate over the Atlantic Ocean (total Nm = 4.612 in Figure 4). Similar results of less genetic differentiation over the American Continents were reported in nuclear microsatellite studies of *Hibiscus pernambucensis* [7] and *Rhizophora mangle* [42], despite the distinctive differentiation for both sea-dispersal plants shown by cpDNA markers.

One possible explanation for the substantial migration between the West Atlantic and East Pacific populations as shown in nuclear DNA markers is pollen dispersal by pollinators over the Isthmus of Panama, as was discussed for *Hibiscus pernambucensis* [7] and New World *Rhizophora* [42]. A large bee, *Xylocopa*, which is a known pollinator of *I. pes-caprae caprae* [44], may occasionally move over the Isthmus of Panama. Another explanation is that the global range was formed before the closure of the Isthmus of Panama and the diversification of nuclear markers was not sufficient to suggest population diversification. However, this is not likely for subsp. *brasiliensis*, because the history of population formation was sufficiently deep to shape the genetic structure between the IWP and AEP observed in this study (Figure 3) despite the lower genetic structure over the American continents. For the same reason, we do not believe that it is likely that migration between the West and East Pacific populations going around the world in the other direction (that is via the Indian Oceanic and West Pacific regions) is sufficient for substantial migration over the American continents. Sea dispersal by drifting seeds around the southern tip of South America is also a possibility. This is also not likely because the direction of the ocean currents on both the Pacific and Atlantic sides of South America are in the opposite direction, preventing dispersal between the 2 regions, but we do not have enough data to discuss this further. To clarify the reason for substantial migration over the American continents, further studies on the history of population expansion of subsp. *brasiliensis* are necessary.

### Different effects of barriers shape the genetic structure of subsp. *brasiliensis*

As was briefly discussed earlier, our study suggests that other land and oceanic barriers known to shape the genetic structure of widespread sea-dispersal plants have different effects on the genetic structure of subsp. *brasiliensis*. A similar genetic structure was superficially suggested by the geographical distribution of the haplotypes of nuclear markers reported in our previous study [12] and in this study (Figure S1), but our statistical analyses enabled us to discuss the differences in better ways.

The East Pacific Ocean and Africa are both strong barriers for many other sea-dispersal plants such as mangroves [5]. Although the two barriers have shaped the genetic structure of subsp. *brasiliensis* and the populations were structured into two clusters almost equivalent to IWP and AEP (Figure 3), the genetic structure was not clearly segregated by the geographic barriers, which also suggests migration over the barriers. The case for the East Pacific is apparent. Three populations (20, 21, and 22 in Figure 3) in the West Pacific around the boundary between the IWP and AEP show admixture patterns, which can be caused by genetic mixture between the two clusters in this area. These results suggest that the East Pacific Ocean shaped the genetic structure of subsp. *brasiliensis*, but the effect was not strong enough to completely prevent long-distance dispersal of *I. pes-caprae* subsp. *brasiliensis* by sea-drifted seeds. Recent studies have also suggested a similar pattern of trans-Pacific dispersal for an AEP mangrove species, *Rhizophora mangle*, which colonized South Pacific islands (where the species is called *Rhizophora samoensis*) from the East Pacific [42].

Another mismatch between genetic clustering and biogeographic boundaries shown in East Africa is more complicated. Two populations (6 and 7 in Figure 3) in East Africa show a slight admixture which also suggests migration between the two clusters in the STRUCTURE analyses, but most of the samples from these populations are in the same cluster with the AEP populations, including those from West Africa, and populations 7 and 8 show clear segregation even though they are geographically close and no apparent geographic barrier exists between them (Figure 1). The results of MIGRATE-N also reflect the genetic structure over the African continent by asymmetric migration: very small westward migration (Nm**_IO to EA_** = 0.511) and much larger eastward migration (Nm**_EA to IO_** = 2.403) (Figure 4 and Table 2). We still do not have any reliable explanations for this asymmetrical migration over Africa and the clear boundary of genetic structure between the East African and Madagascar populations. The effects of modern ocean currents may not be the reason, because there are strong southwestward currents through the straight between East Africa and Madagascar, and no apparent currents from West Africa to East Africa. Historical effects on genetic structure such as contraction of the population to refugia in glacial periods followed by a strong bottleneck and range expansion, or genetic influence from closely related species via hybridization might have caused these kinds of genetic structures, but further studies are necessary to answer the question.

The other three possible barriers for sea-dispersal plants (Figure 4 and Table 2), the Malay Peninsula region, American continents, and Atlantic Ocean, have not prevented substantial trans-barrier migration among populations of subsp. *brasiliensis*. The Malay Peninsula region is known to shape distinct genetic structures in some mangrove plants between the Indian Ocean and West Pacific regions [5], [45], but for subsp. *brasiliensis*, the migration between the two regions was the highest among the population pairs studied (Figure 4 and Table 2). A considerable level of migration over the Malay Peninsula was also reported in *Hibiscus tiliaceus*, which is distributed from West Africa to the South Pacific [6], [7]. These studies suggest that the Malay Peninsula has different levels of effectiveness as a barrier to migration for different groups of sea-dispersal plants, which is perhaps related to their seed dispersal ability, the strength of ocean currents between the two regions, or the history of populations. Because *I. pes-caprae* subsp. *brasiliensis* retains seed buoyancy for more than 3 months [11], it can be substantially dispersed over the barrier by ocean currents, unlike mangroves like *Bruguiera gymnorhiza* [45]. Further studies on the seed buoyancy and viability of the sea-drifted seeds of these species and the history of population divergence are necessary.

### The strong genetic difference between two subspecies of *Ipomoea pes-caprae*

*Ipomoea pes-caprae* subsp. *pes-caprae* and subsp. *brasiliensis* have a distributional boundary where no apparent land or oceanic barriers exist. Taxonomists have considered that the differences of the two subspecies in gross morphology and distribution ranges were at the subspecies level [9], [10]. However, the present study showed that the two subspecies are clearly differentiated genetically and the genetic differences between them were considerably high (Nm<1), which could cause strong differentiation by genetic drift according to Kimura and Maruyama [41]. This suggests that the two subspecies may have cryptic ocean barriers that prevent migration by sea dispersal between the distribution ranges, and/or they have experienced historical differentiation that caused local adaptation to different environmental factors in each region. Further oceanographic studies as well as differentiation of environmental factors will be necessary.

## Conclusion

In this study, using samples from populations that cover the distribution range of *I. pes-caprae*, coalescent-based approaches suggested that migration among distant populations of subsp. *brasiliensis* was maintained across its global range. Long-distance dispersal by sea-drifted seeds is strong enough for the species to maintain migration between distant populations connected by the sea. Perhaps the effective seed dispersal and the similar ecological environment at tropical beaches may allow the colonization of subsp. *brasiliensis* across the pantropical range. Another important finding is the substantial level of migration over the American continents, which are an apparent land barrier for sea dispersal, and this might be due to trans-isthmus pollen dispersal. Thus, the migration and gene flow among populations across the vast range of *I. pes-caprae* is maintained not only by seed dispersal by sea-drifted seeds, but also by pollen flow over the American continents.

## Supporting Information

Figure S1 **Geographical distribution of nuclear DNA haplotypes of** ***Ipomoea pes-caprae*** **.** Each circle represents one population including eight individuals. Colors used in pie charts represent haplotypes shown in Figure S2.
(TIF)

Figure S2 **Haplotype networks of five low copy nuclear genes of** ***Ipomoea pes-caprae*** **.** Each haplotype is shown as a circle, the size of which is proportional to the number of individuals that have the haplotype. A small open circle connecting haplotypes represents a mutational step between the haplotypes.
(TIF)

Figure S3 **STRUCTURE analysis of** ***Ipomoea pes-caprae*** **populations from K = 2 to K = 6.** Vertical bars represent the membership coefficients (Q) of individual plants. The horizontal axes correspond to the regional grouping of populations. Left to right: subsp. *pes-caprae*, Indian Ocean, West Pacific, East Pacific, West Atlantic, and East Atlantic regions. Numbers at the upper side designate the population as are shown in Figure 1 and Table S1.
(TIF)

Table S1 **Geographic location and sample size of Ipomoea pes-caprae populations in this study.**
(DOC)

Table S2 **List of primers used for sequencing of nuclear regions in this study.**
(DOC)

Table S3 **Summary of Genetic Divergences and Differentiations between each two regions.**
(PDF)

Table S4 **Population pairwise average Fst for Ipomoea pes-caprae calculated rom seven low-copy gene sequences.** Population pairs within a rectangler of dotted lines show the ones within a geographic region employed in this study (names of regions are shown in gray letters).
(PDF)

Table S5 **Maximum likelihood estimates (MLE), 95% confidence interval of θ and migration rates (M) and number of migrants (Nm) obtained from MIGRATE-N.**
(PDF)
