# Supplementary figures and images for: Long-Distance Dispersal by Sea-Drifted Seeds Has Maintained the Global Distribution of Ipomoea pes-caprae subsp. brasiliensis (Convolvulaceae)
Source: PLoS One. 2014 Apr 22;9(4):e91836. doi: 10.1371/journal.pone.0091836 (PMC3995641; doi:10.1371/journal.pone.0091836)

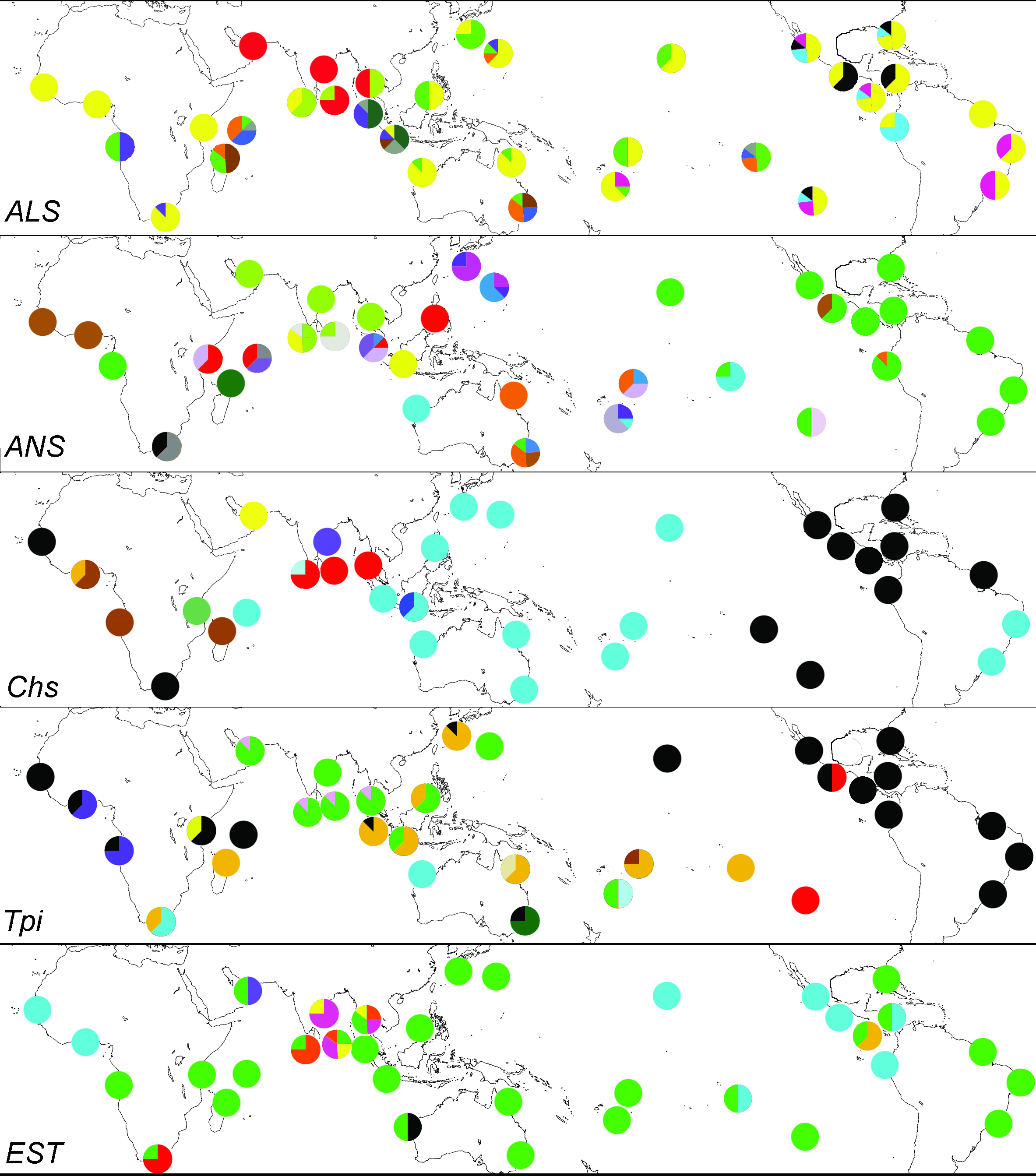

Supplement: Figure S1 — Geographical distribution of nuclear DNA haplotypes of Ipomoea pes-caprae . Each circle represents one population including eight individuals. Colors used in pie charts represent haplotypes shown in Figure S2. (TIF) [file pone.0091836.s001.tif]

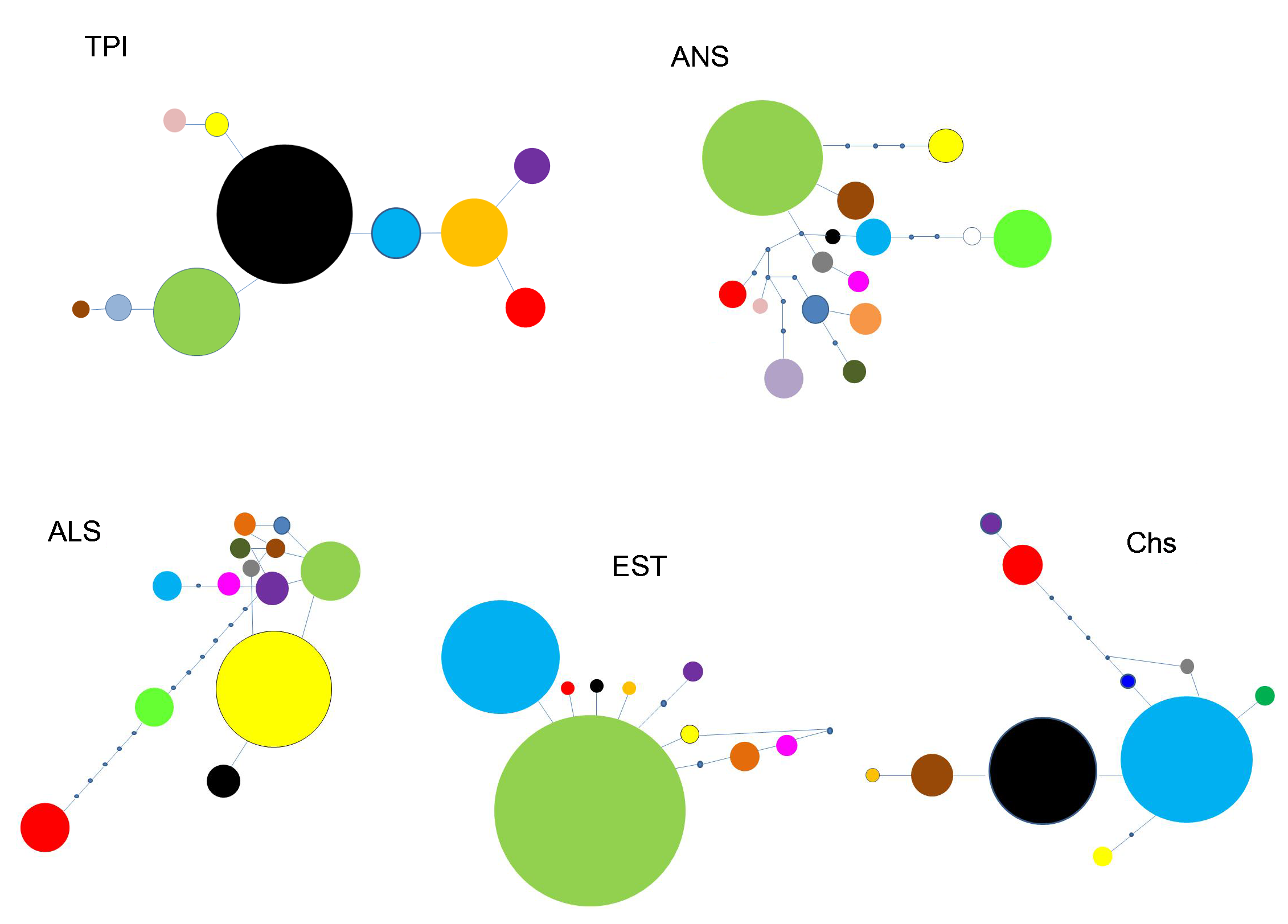

Supplement: Figure S2 — Haplotype networks of five low copy nuclear genes of Ipomoea pes-caprae . Each haplotype is shown as a circle, the size of which is proportional to the number of individuals that have the haplotype. A small open circle connecting haplotypes represents a mutational step between the haplotypes. (TIF) [file pone.0091836.s002.tif]

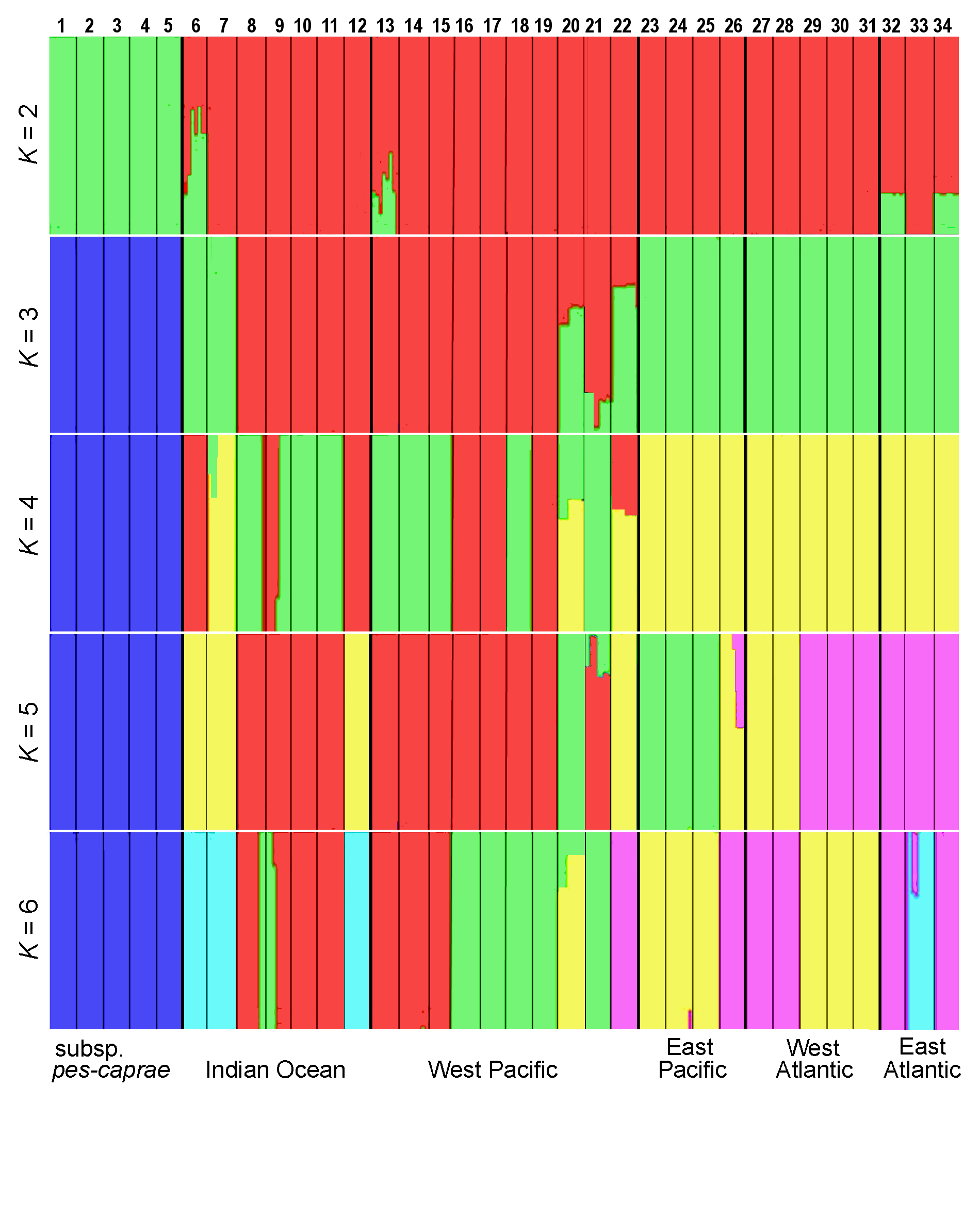

Supplement: Figure S3 — STRUCTURE analysis of Ipomoea pes-caprae populations from K = 2 to K = 6. Vertical bars represent the membership coefficients (Q) of individual plants. The horizontal axes correspond to the regional grouping of populations. Left to right: subsp. pes-caprae, Indian Ocean, West Pacific, East Pacific, West Atlantic, and East Atlantic regions. Numbers at the upper side designate the population as are shown in Figure 1 and Table S1. (TIF) [file pone.0091836.s003.tif]
