# Supplementary material for: Long-Distance Dispersal by Sea-Drifted Seeds Has Maintained the Global Distribution of Ipomoea pes-caprae subsp. brasiliensis (Convolvulaceae)
Source: PLoS One. 2014 Apr 22;9(4):e91836. doi: 10.1371/journal.pone.0091836 (PMC3995641; doi:10.1371/journal.pone.0091836)
Supplement: Table S1 — Geographic location and sample size of Ipomoea pes-caprae populations in this study. (DOC) [file pone.0091836.s004.doc]

| No. | Oceanic Region Population | Country | Geographic  region | Sample size | Latitude | Longitude |
| --- | --- | --- | --- | --- | --- | --- |
|  | **Indian Ocean** |  |  |  |  |  |
|  | ***I . pes-caprae* (L.) R. Br. subsp. *pes-caprae* (L.) Ooststr.** |  |  |  |  |  |
| 1 | Fujaira | UAE | IO | 8 | 25.493 | 56.361 |
| 2 | Wattala. Negambo | Sri Lanka | IO | 8 | 6.997 | 79.873 |
| 3 | Hambantoa | Sri Lanka | IO | 8 | 6.135 | 81.135 |
| 4 | Maharastra | India | IO | 8 |  |  |
| 5 | Phuket: Kmala Beach | Thailand | IO | 8 | 7.807 | 98.299 |
|  | ***I. pes-caprae* (L.) R. Br. subsp. *brasiliensis* (L.) Ooststr.** |  |  |  |  |  |
|  |  |  |  |  |  |  |
| 6 | Tegula river mouth | South Africa | IO | 8 | -29.23 | 31.487 |
| 7 | Mjimwema | Tanzania | IO | 8 | -6.836 | 39.323 |
| 8 | Sambava | Madagascar | IO | 8 | -14.295 | 50.189 |
| 9 | Anse A La Manche, Mahe Island | Seychelles | IO | 8 | -4.961 | 55.592 |
| 10 | Southwest of Changi Airport | Singapore | IO | 8 | 1.317 | 103.981 |
| 11 | Pantai Tratae, Java | Indonesia | IO | 8 | -6.989 | 106.381 |
| 12 | Fucarne I., Port Headlamd, West Australia | Australia | IO | 8 | -20.307 | 118.584 |
|  | **West Pacific** |  |  |  |  |  |
| 13 | Seashore of Tak Doan, 35 km from Puerto Princesa | Philippine | WP | 8 | 9.461 | 118.587 |
| 14 | Takanabe, Miyazaki Pref. | Japan | WP | 8 | 32.161 | 131.534 |
| 15 | Ogasawara I. Tokyo | Japan | WP | 8 | 26.609 | 142.179 |
| 16 | N. of Ellis Beach, Queensland | Australia | WP | 8 | -16.717 | 145.635 |
| 17 | Harrington, Crowdy Head, New South Wales | Australia | WP | 8 | -31.849 | 152.748 |
| 18 | Haashini-Lavengatonga, Sopu | Tonga | WP | 8 | -21.12 | -175.229 |
| 19 | NW seashore, Upolu | Samoa | WP | 8 | -13.863 | -171.694 |
| 20 | Oahu Isl., Hawaii | U.S.A. | WP | 8 | 21.367 | -157.711 |
| 21 | Anaho, Nuku Hiva, Marquesie Isl. | French Polynesia | WP | 8 | -9.951 | -139.049 |
| 22 | Tongaliki. Easter Isl. | Chile | WP | 8 | -27.122 | -109.175 |
|  | **East Pacific** |  |  |  |  |  |
| 23 | Playa Majahual, Sinaloa | Mexico | EP | 8 | 22.797 | -105.959 |
| 24 | Loca Blanca, Oaxaca | Mexico | EP | 8 | 15.709 | -96.718 |
| 25 | Esmeralda | Ecuador | EP | 8 | 0.958 | -79.709 |
| 26 | Vera Cruz | Panama | EP | 8 | 8.915 | -79.568 |
|  | **West Atlantic** |  |  |  |  |  |
| 27 | Cuango, Colon | Panama | WA | 8 | 9.392 | -79.871 |
| 28 | Indian R., Florida | U.S.A. | WA | 8 | 27.872 | -82.851 |
| 29 | Praia do Crispin, Para | Brazil | WA | 8 | -0.647 | -47.562 |
| 30 | Barra de Sirinhaem, Pernanbuco | Brazil | WA | 8 | -8.618 | -35.06 |
| 31 | Recreio, Rio de Janeiro | Brazil | WA | 8 | -22.996 | -43.325 |
|  | **East Atlantic** |  |  |  |  |  |
| 32 | Joal-Fadiout | Senegal | EA | 8 | 14.256 | -16.904 |
| 33 | Labadi Beach | Ghana | EA | 8 | 5.583 | -0.106 |
| 34 | Musul. Luanda | Angola | EA | 8 | -8.856 | 13.199 |

Table S1. Geographic location and sample size of *Ipomoea pes-caprae* populations in this study
