# Supplementary material for: Long-Distance Dispersal by Sea-Drifted Seeds Has Maintained the Global Distribution of Ipomoea pes-caprae subsp. brasiliensis (Convolvulaceae)
Source: PLoS One. 2014 Apr 22;9(4):e91836. doi: 10.1371/journal.pone.0091836 (PMC3995641; doi:10.1371/journal.pone.0091836)
Supplement: Table S2 — List of primers used for sequencing of nuclear regions in this study. (DOC) [file pone.0091836.s005.doc]

| **Primer** | **Length (bp)** | **Recombination sites** | **Exon** | **Intron** | **Primer sequence (5'->3')** |
| --- | --- | --- | --- | --- | --- |
| *AlS* | 430-448 | 68-86 | 1-430 | - | F- ACTCATCAAGAACCTGAATAGCATA |
|  |  |  |  |  | R-GGATTCCTGTGGCTAGTACTTTAAT |
| *ANS* | 680-699 | 164-171 | 386-680 | 1-385 | F-TTTGGGTAGTAGTTTATCTTCATTT |
|  |  |  |  |  | R-GTATTATAGGTTCAAACCACAGAGT |
| *Chs* | 738-751 | 691-703 | 150-548, 703-738 | 1-150, 548-703 | F-CTTTACCAAACAATCCTCTACGTTT |
|  |  |  |  |  | R-TATCTGAAATAACCCATTTAGTGTT |
| *EST* | 425 | - | 1-397 | - | F-TGTAGTTACTCACCTCTTTCTTTCT |
|  |  |  |  |  | R-AATATTGTTTTTGTTTTTCTCCTTA |
| *HSP* | 785 | - | 1-40, 481-785 | 40-481 | F-TAACGTATTATGTATTTGCACTGAT |
|  |  |  |  |  | R-TTTAATTACAGAACCAGAATTACCT |
| *TPI* | 443 | - | 1-440 | - | F-GTTGGTGAAACACTTGAGCAGAGAG |
|  |  |  |  |  | R-GACAATTTTAGACCAATCTGATACT |
| *Waxy* | 574-576 | - | 1-46, 126-301, 405-574 | 47-125, 302-404 | F-GCTCATTTGGAATTTTATGC |
|  |  |  |  |  | R-AAATCAGCACCAGCAGTAAT |

**Table S2. List of primers used for sequencing of nuclear regions in this study.**
