# Supplementary material for: Long-Distance Dispersal by Sea-Drifted Seeds Has Maintained the Global Distribution of Ipomoea pes-caprae subsp. brasiliensis (Convolvulaceae)
Source: PLoS One. 2014 Apr 22;9(4):e91836. doi: 10.1371/journal.pone.0091836 (PMC3995641; doi:10.1371/journal.pone.0091836)
Supplement: Table S3 — Summary of Genetic Divergences and Differentiations between each two regions. (PDF) [file pone.0091836.s006.pdf]

Table S3. Summary of Genetic Divergences and Differentiations between each two regions

Locus	Pair	S	Sf	Ktotal	Knc	Ks	Ka	Ka/Ks
ALS	*pes-caprae vs brasiliensis*	17	0	0.02445	0.00000	0.02993	0.00590	0.19713
ANS	*pes-caprae vs brasiliensis*	26	0	0.01550	0.02061	0.00904	0.00000	0.00000
CHS	*pes-caprae vs brasiliensis*	30	0	0.01502	0.00000	0.01928	0.00231	0.11981
EST-2	*pes-caprae vs brasiliensis*	7	0	0.00443	0.00000	0.01147	0.00246	0.21447
HSP-90	*pes-caprae vs brasiliensis*	5	0	0.00323	0.00000	0.00490	0.00280	0.57143
TPI	*pes-caprae vs brasiliensis*	4	0	0.00374	0.00000	0.00625	0.00306	0.48960
Waxy	*pes-caprae vs brasiliensis*	4	0	0.00233	0.00000	0.00000	0.00293	0.00000
Average	*pes-caprae vs brasiliensis*			0.00981	0.00294	0.01155	0.00278	0.22749
ALS	East Pacific vs West Atlantic	5	0	0.00426	0.00000	0.00434	0.00409	0.94240
ANS	East Pacific vs West Atlantic	7	0	0.00111	0.00171	0.00095	0.00000	0.00000
CHS	East Pacific vs West Atlantic	1	0	0.00054	0.00000	0.00072	0.00000	0.00000
EST-2	East Pacific vs West Atlantic	2	0	0.00226	0.00000	0.00290	0.00000	0.00000
HSP-90	East Pacific vs West Atlantic	3	0	0.00339	0.00000	0.00339	0.00000	0.00000
TPI	East Pacific vs West Atlantic	3	0	0.00221	0.00000	0.00284	0.00000	0.00000
Waxy	East Pacific vs West Atlantic	3	0	0.00129	0.00000	0.00162	0.00000	0.00000
Average	East Pacific vs West Atlantic			0.00215	0.00024	0.00239	0.00058	0.13463
ALS	Indian vs Atlantic	7	0	0.00330	0.00000	0.00389	0.00135	0.34704
ANS	Indian vs Atlantic	17	0	0.00627	0.00984	0.00080	0.00000	0.00000
CHS	Indian vs Atlantic	20	0	0.01922	0.00000	0.02385	0.00525	0.22013
EST-2	Indian vs Atlantic	4	0	0.00121	0.00000	0.00124	0.00110	0.88710
HSP-90	Indian vs Atlantic	5	0	0.00155	0.00000	0.00187	0.00147	0.78610
TPI	Indian vs Atlantic	6	0	0.00322	0.00000	0.00372	0.00149	0.40054
Waxy	Indian vs Atlantic	3	0	0.00365	0.00000	0.00459	0.00000	0.00000
Average	Indian vs Atlantic			0.00549	0.00141	0.00571	0.00152	0.37727
ALS	Indian vs West Pacific	17	0	0.00280	0.00000	0.00361	0.00259	0.71745
ANS	Indian vs West Pacific	26	0	0.00729	0.01088	0.00550	0.00088	0.16000
CHS	Indian vs West Pacific	20	0	0.00910	0.00000	0.01136	0.00232	0.20423
EST-2	Indian vs West Pacific	7	0	0.00042	0.00000	0.00110	0.00023	0.20909
HSP-90	Indian vs West Pacific	5	0	0.00186	0.00000	0.00403	0.00129	0.32010
TPI	Indian vs West Pacific	4	0	0.00260	0.00000	0.00293	0.00149	0.50853
Waxy	Indian vs West Pacific	4	0	0.00185	0.00000	0.00233	0.00000	0.00000
Average	Indian vs West Pacific			0.00370	0.00155	0.00441	0.00126	0.30277

The parameters shown are sequence length (bp), the number of sequences analyzed (N), the number of polymorphisms specific to each region (Sx), nucleotide diversityof all sites (pTotal) and that of noncoding regions (pnc) and synonymous (ps) and nonsynonymous (DTotal), noncodingsites (pa), and Tajima's D for all sites regions (Dnc),synonymous (Ds), and nonsynonymous sites (Da), and Fay's H (H) tests.
