# Supplementary material for: Long-Distance Dispersal by Sea-Drifted Seeds Has Maintained the Global Distribution of Ipomoea pes-caprae subsp. brasiliensis (Convolvulaceae)
Source: PLoS One. 2014 Apr 22;9(4):e91836. doi: 10.1371/journal.pone.0091836 (PMC3995641; doi:10.1371/journal.pone.0091836)
Supplement: Table S4 — Population pairwise average Fst for Ipomoea pes-caprae calculated rom seven low-copy gene sequences. Population pairs within a rectangler of dotted lines show the ones within a geographic region employed in this study (names of regions are shown in gray letters). (PDF) [file pone.0091836.s007.pdf]

Table S4. Population pairwise average Fst for Ipomoea pes-caprae calculated rom seven low-copy gene sequences.
Population pairs within a rectangler of dotted lines show the ones within a geographic region employed in this study (names of regions are shown in gray letters)

	1	2	3	4	5	6	7	8	9	10	11	12	13	14	15	16	17	18	19	20	21	22	23	24	25	26	27	28	29	30	31	32	33	34
1. UAE					pes-caprae																													
2. Sri Lanka A	0.45																																	
3. Sri Lanka B	0.42	0.25																																
4. India	0.53	0.46	0.28																															
5 Thailand	0.50	0.18	0.26	0.44																														
6. South Africa	0.68	0.71	0.58	0.75	0.71							IO																						
7. Tanzania	0.60	0.63	0.52	0.69	0.62	0.48																												
8. Madagascar	0.87	0.90	0.81	0.95	0.89	0.73	0.72																											
9. Seychelles	0.73	0.77	0.62	0.80	0.77	0.68	0.58	0.54																										
10. Singapore	0.85	0.88	0.75	0.91	0.87	0.59	0.65	0.26	0.36																									
11. Indonesia, Java	0.72	0.76	0.62	0.82	0.75	0.55	0.55	0.50	0.42	0.34																								
12 Australia, W.	0.88	0.90	0.79	0.97	0.90	0.48	0.69	0.73	0.53	0.50	0.58																							
13. Philippines	0.75	0.78	0.67	0.83	0.77	0.51	0.50	0.51	0.36	0.44	0.42	0.55										WP												
14. Japan, Miyazaki	0.83	0.87	0.73	0.92	0.87	0.58	0.68	0.47	0.21	0.29	0.39	0.47	0.32																					
15. Japan, Ogasawara	0.74	0.78	0.64	0.81	0.77	0.56	0.54	0.51	0.23	0.50	0.50	0.60	0.20	0.31																				
16. Australia, QL	0.86	0.89	0.78	0.94	0.88	0.46	0.60	0.41	0.70	0.46	0.55	0.70	0.44	0.62	0.43																			
17. Australia, NSW	0.84	0.88	0.75	0.93	0.87	0.72	0.71	0.56	0.19	0.38	0.46	0.53	0.46	0.27	0.38	0.74																		
18. Tonga	0.77	0.81	0.68	0.85	0.81	0.56	0.52	0.54	0.31	0.47	0.49	0.57	0.19	0.33	0.12	0.47	0.39																	
19. Samooa	0.85	0.88	0.76	0.91	0.87	0.51	0.62	0.40	0.52	0.24	0.44	0.56	0.52	0.37	0.58	0.23	0.50	0.55																
20. USA. Hawaii	0.79	0.80	0.67	0.84	0.81	0.65	0.72	0.79	0.28	0.57	0.61	0.55	0.51	0.37	0.38	0.82	0.47	0.44	0.64															
21. FP, Marquesus	0.88	0.89	0.77	0.94	0.90	0.52	0.76	0.46	0.70	0.47	0.59	0.73	0.53	0.63	0.59	0.43	0.73	0.58	0.49	0.78														
22. Chile, Easter	0.87	0.90	0.77	0.94	0.89	0.56	0.55	0.58	0.53	0.49	0.53	0.62	0.30	0.49	0.35	0.50	0.58	0.34	0.60	0.66	0.60													
23. Mexico, SIN	0.80	0.79	0.67	0.83	0.80	0.58	0.63	0.89	0.68	0.81	0.68	0.86	0.55	0.80	0.53	0.69	0.87	0.59	0.79	0.57	0.53	0.49				EP								
24. Mexico, OAX	0.80	0.80	0.67	0.83	0.80	0.57	0.62	0.91	0.69	0.83	0.69	0.84	0.54	0.81	0.53	0.68	0.89	0.58	0.79	0.43	0.49	0.50	0.12											
25 Ecuador	0.79	0.79	0.67	0.82	0.79	0.64	0.70	0.91	0.71	0.83	0.71	0.91	0.61	0.85	0.58	0.75	0.89	0.65	0.85	0.53	0.51	0.51	0.16	0.07										
26 Panama, VER	0.76	0.77	0.66	0.81	0.78	0.58	0.60	0.86	0.65	0.79	0.65	0.83	0.52	0.78	0.49	0.65	0.83	0.55	0.77	0.60	0.49	0.44	0.17	0.14	0.17									
27. Panama, COL	0.82	0.84	0.72	0.88	0.84	0.48	0.60	0.80	0.72	0.72	0.61	0.74	0.50	0.72	0.55	0.58	0.81	0.55	0.70	0.65	0.46	0.38	0.21	0.19	0.28	0.13					WA			
28. USA, Florida	0.76	0.78	0.65	0.83	0.78	0.54	0.47	0.77	0.56	0.70	0.56	0.78	0.41	0.68	0.38	0.53	0.75	0.44	0.65	0.58	0.50	0.35	0.13	0.14	0.22	0.12	0.14							
29. Brazil, PA	0.62	0.65	0.53	0.69	0.65	0.40	0.32	0.81	0.59	0.74	0.58	0.78	0.45	0.74	0.40	0.53	0.78	0.47	0.70	0.61	0.68	0.39	0.31	0.30	0.40	0.33	0.33	0.15						
30. Brazil, PE	0.76	0.78	0.64	0.82	0.78	0.63	0.51	0.76	0.41	0.54	0.47	0.66	0.55	0.53	0.53	0.70	0.60	0.60	0.51	0.45	0.64	0.50	0.28	0.29	0.35	0.31	0.33	0.15	0.33					
31. Brazil, RJ	0.76	0.78	0.64	0.82	0.78	0.65	0.53	0.77	0.41	0.53	0.48	0.68	0.57	0.53	0.55	0.73	0.60	0.61	0.53	0.46	0.64	0.50	0.28	0.29	0.35	0.32	0.34	0.17	0.35	0.00				
32. Senegal	0.81	0.81	0.70	0.84	0.81	0.55	0.62	0.96	0.75	0.89	0.74	0.83	0.60	0.87	0.55	0.68	0.93	0.62	0.85	0.61	0.55	0.56	0.30	0.16	0.20	0.26	0.36	0.29	0.43	0.47	0.49			EA
33. Ghana	0.80	0.80	0.72	0.84	0.80	0.59	0.62	0.72	0.79	0.76	0.71	0.72	0.72	0.76	0.73	0.70	0.84	0.73	0.73	0.64	0.61	0.72	0.65	0.50	0.55	0.58	0.60	0.64	0.67	0.67	0.69	0.49		
34. Angola	0.72	0.74	0.64	0.79	0.73	0.59	0.58	0.54	0.67	0.60	0.61	0.82	0.66	0.62	0.67	0.72	0.73	0.67	0.63	0.75	0.71	0.63	0.70	0.72	0.76	0.75	0.66	0.60	0.50	0.57	0.57	0.93	0.57	
