# Supplementary material for: Long-Distance Dispersal by Sea-Drifted Seeds Has Maintained the Global Distribution of Ipomoea pes-caprae subsp. brasiliensis (Convolvulaceae)
Source: PLoS One. 2014 Apr 22;9(4):e91836. doi: 10.1371/journal.pone.0091836 (PMC3995641; doi:10.1371/journal.pone.0091836)
Supplement: Table S5 — Maximum likelihood estimates (MLE), 95% confidence interval of θ and migration rates (M) and number of migrants (Nm) obtained from MIGRATE-N. (PDF) [file pone.0091836.s008.pdf]

Table 5_Maximum likelihood estimates (MLE), 95% confidence interval of θ and migration rates (M) and number of migrants (Nm) obtained from MIGRATE-N

	0.05	MLE	0.95	Nm= Mθ/4
$\theta_{pes\text{-}aprae}$	0.00225	0.00443	0.00640	
θ_{In}	0.00130	0.00230	0.00480	
θ_{WP}	0.00182	0.00310	0.00630	
θ_{EP}	0.00150	0.00300	0.00480	
θ_{WA}	0.00146	0.00230	0.00310	
θ_{EA}	0.00045	0.00153	0.00630	
$M_{pescaprae>brasiliensis}$	105.0	135.7	162.0	0.259
$M_{brasiliensis>pescaprae}$	125.0	180.8	470.6	0.565
$M_{In>WP}$	1009.5	1236.3	2309.2	2.794
$M_{WP>In}$	1863.0	2133.3	3903.7	6.246
$M_{WP>EP}$	909.0	1176.2	2007.2	1.566
$M_{EP>WP}$	821.0	1119.8	1893.5	2.291
$M_{EP>WA}$	1182.5	1569.2	1904.8	2.286
$M_{WA>EP}$	2283.8	2956.3	4989.7	3.892
$M_{WA>EA}$	2073.6	3002.2	3487.5	2.703
$M_{EA>WA}$	659.0	863.7	964.4	1.519
$M_{EA>In}$	636.0	817.8	1501.9	2.403
$M_{In>EA}$	4.9	153.5	426.3	0.512

Regional groups (WA: West Atlantic, EA: East Atlantic, In: Indian, WP: West Pacific, EP: East Pacific).
